# Sensitivity of Piezoelectric Stack Actuators

**DOI:** 10.3390/s23239542

**Published:** 2023-11-30

**Authors:** Xishan Jiang, Jing Zheng, Ning Wang, Jie Pan

**Affiliations:** 1 Department of Instrument Science and Engineering, Zhejiang University, Hangzhou 310027, China; jxishan@zju.edu.cn (X.J.); 10915008@zju.edu.cn (J.Z.); zjuningwang@163.com (N.W.); 2 Department of Mechanical Engineering, University of Western Australia, Crawley, WA 6009, Australia

**Keywords:** piezoelectric stack actuator, nonlinear hysteresis effect, sensitivity analysis

## Abstract

This paper investigates the properties of a mass−attached piezoelectric stack actuator and analyzes its sensitivity, which is defined as the spectrum of the driving force (the output) caused by a single−frequency voltage (the input). The force spectrum is utilized because of the nonlinear hysteresis effect of the piezoelectric stack. The sensitivity analysis shows that the nonlinear dynamics of the actuator can be interpreted as a cascade of two subsystems: a nonlinear hysteresis subsystem and a linear mechanical subsystem. Analytical solutions of the nonlinear differential equations are proposed, which show that the nonlinear transformation can be described by a steady−state mapping of a single−frequency voltage input to a multiple−frequency driving force at the driving frequency and its odd harmonics. The steady−state sensitivity is then determined by the response of the mechanical subsystem to the line spectrum of the driving force. The maximum sensitivity can be achieved by setting the frequency of the input voltage close to the natural frequency of the mechanical subsystem. The analytical model is also validated by a numerical model and experimental results and it may be used for the analysis and design of piezoelectric actuators with different structural configurations.

## 1. Introduction

Piezoelectric ceramics are typical smart materials that are capable of actuation. They can be used to convert electrical energy into mechanical energy via their inverse piezoelectric effect and can function as actuators to generate or control a response. Their piezoelectric features have found numerous applications in the fields of vibration control and condition monitoring of micro−/nano−positioning platforms and structural vibration [1].

The piezoelectric ceramics are usually stacked to obtain a larger displacement and force, with a lower voltage input. This stacked structure is equivalent to each layer of piezoelectric materials being connected in series mechanically and connected in parallel in the circuit [2]. Therefore, they can withstand high pressure and provide the highest stiffness of all piezo−actuator designs and have been widely used for both static and dynamic operation [3]. However, due to nonlinear hysteresis, piezoelectric stack actuators produce errors in open−loop control systems. The hysteresis characteristic is an inherent feature of piezoelectric materials. During the process of increasing and decreasing the input voltage, the output displacements of piezoelectric ceramics may not be along the same path. In other words, the output of the actuator depends not only on the instantaneous input but also on the history of its output [4]. This nonlinear characteristic of the material itself hinders high−precision positioning and control and reduces the performance of control systems. The compensation and control of hysteresis errors often require accurate models of piezoelectric stack actuators.

A lot of work has been devoted to the modeling of the nonlinear hysteresis of piezoelectric ceramics. The models may be divided into physics−based models and phenomenological models. The physical models, such as the Jiles−Atherton model [5], were constructed using the electromagnetic−force coupling relationship from the perspective of the microscopic mechanisms of hysteresis in the material. The parameters of the model and their complex relationships with the system output pose difficulties for parameter identification, which limit the application of such models. On the other hand, the mathematical models describe the hysteresis phenomenon based on the input and output relationship of the actuator. This model is also known as the phenomenological model, including (1) differential equation models, such as the Bouc−Wen model [6,7] and Duhem model [8]; (2) operator models, such as the Preisach model [9], Krasnosel’skii−Pokrovskii model [10], and Prandtl−Ishlinskii model [11]; and (3) intelligent computing models, such as artificial neural networks [12]. The Bouc−Wen hysteresis model has been widely used to model hysteresis with promising results. The model was first proposed by Bouc and then improved by Wen for describing the hysteresis phenomenon. It was used to simulate hysteresis curves of different shapes and types. It requires only a few parameters and has been extensively used for modeling piezoelectric actuators in various control systems.

Due to the advantages of the Bouc−Wen model represented by nonlinear differential equations, the coupled models based on it can be implemented to study piezoelectric stack actuators and piezoelectric stack−based systems, as they can not only represent the hysteresis mathematically but also describe the dynamics [13,14]. Furthermore, Ha et al. [15] and Gomis−Bellmunt et al. [16] proved that the hysteretic characteristics of piezoelectric stack actuators can be modeled by using the Bouc−Wen hysteresis operator and considering the piezoelectric stack actuators as a single−degree−of−freedom (DOF) mass−spring−damper system. This second−order system is gaining increasing numbers of applications.

The final step to designing an actuator is to use an appropriate metric to evaluate its performance. Traditionally, the output force of the actuator was evaluated separately without considering the excitation input. Adriaens et al. described the influence of the mechanism on the overall behavior of a piezoelectric stack actuator based on a transfer−function representation [17]. However, the output force due to voltage input could not be represented by another transfer function because of the inherent nonlinear hysteresis property in generating the force [18]. Furthermore, there has been relatively little research in this area. A clearer way to express the relationship between the output force and input voltage of piezoelectric actuators is needed.

Here, the sensitivity of the actuator is defined by a single−frequency voltage as the input and the spectrum of the transmitted force by the actuator as the output. It is found that the nonlinear transformation from the input voltage to the driving force in the actuator has an interesting steady−state mapping from a single−frequency input voltage to a multiple−frequency driving force at the driving frequency and its odd harmonics. As a result, the steady−state sensitivity can be determined by the response of the linear mechanical system of the actuator to the steady−state driving force. The properties of this steady−state hysteretic displacement are further expressed by analytical expressions via approximately solving the first five frequency components of the Bouc−Wen model. Despite the approximation, the analytical expression of the steady−state frequency components of the piezoelectric driving force provides useful explanations of the mechanisms involved in the nonlinear hysteresis. The effect of the model parameters on the accuracy of the model is also discussed.

This paper focuses on the nonlinear input/output relationship of a piezoelectric stack actuator. Firstly, the model of the actuator attached with a proof mass and the definition of the sensitivity are proposed in Section 2. Secondly, analytical solutions of the nonlinear differential equations and the steady−state sensitivity are analyzed in Section 3. Finally, to validate the proposed model, lab experiments are conducted to obtain the vibration of a piezoelectric stack actuator in Section 4. The results from the analytical model, numerical model, and lab experiments show good agreements. The models and results presented in this paper may be used for the analysis and design of piezoelectric actuators with different structural configurations. In addition, the definition of sensitivity we raised in this paper could be used to evaluate the output performance of actuators accurately.

## 2. Problem Description

**Figure 1:**
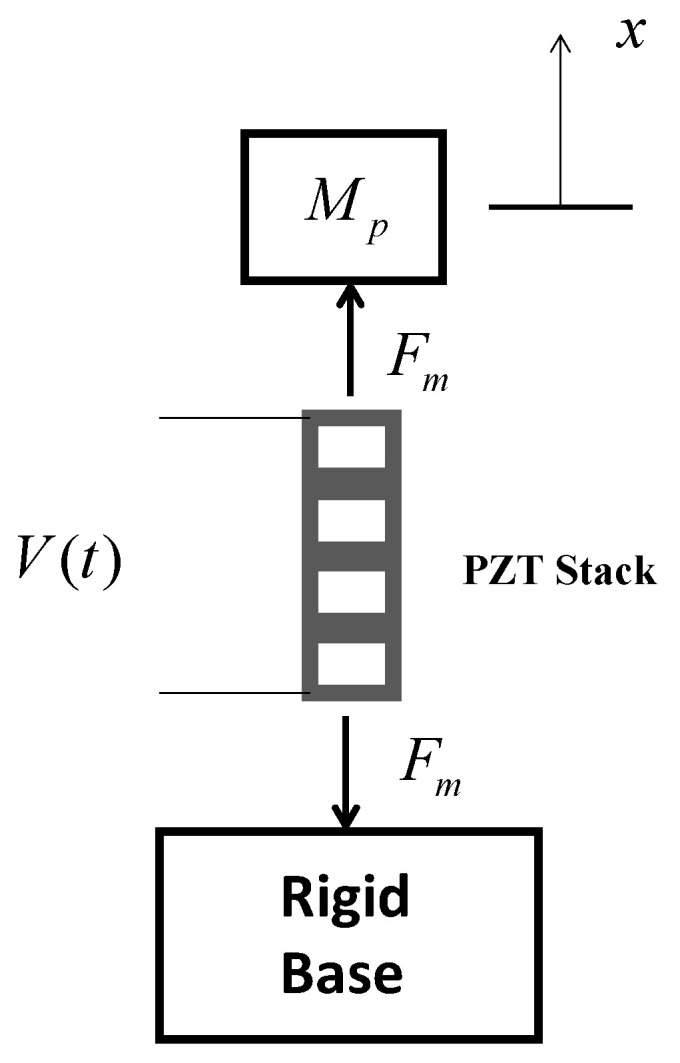
Diagram of a piezoelectric stack actuator between a proof mass and a rigid base.

Figure 1 shows a diagram of a piezoelectric stack actuator driven by a time−dependent sinusoidal voltage V(t) and situated between a proof mass $M_{p}$ on the top of the stack and a rigid base structure on the bottom.

The dynamics of the actuator is described by [19]:

(1) $$(M_{p}+m_{p})\ddot{x}+b_{p}\dot{x}+k_{p}x=k_{p}(cV-h),$$

where x(t) is the displacement of the actuator.$m_{p}$, $b_{p}$, and $k_{p}$ are respectively the mass, damping, and stiffness constants of the piezoelectric stack, and c is the piezoelectric constant. The right−hand side of Equation (1) is defined as the driving force of the piezoelectric actuator, where the hysteresis−induced displacement *h* is described by the first−order nonlinear model [7,19]:

(2) $$\dot{h}=\alpha \dot{V}-\beta \left|\dot{V}\right|h-\gamma \dot{V}\left|h\right|,$$

where α, β, and γ are the constants affected by the shape of the hysteresis curve for the piezoelectric stack. Equations (1) and (2) indicate that the dynamics of the actuator can be divided into two parts. The first part includes the piezoelectric force generation dynamics, whilst the second part includes the oscillator dynamics consisting of the proof mass, stiffness, and damping of the piezoelectric stack. The nonlinear dynamics of the first part allow the generation of other frequency components even though the input voltage is at a single frequency.

The sensitivity of the actuator describes its capability in producing the force output (transmitted force to the base structure) for a given voltage input. In this paper, the sensitivity of the actuator is examined using a single−frequency voltage as the input and the force response at the solid base as the output. Such sensitivity analysis of the nonlinear actuator is motivated by the need for the active acoustical control of structures, such as power transformers and electrical inductors, whereas primary vibration is often dominated by single frequencies. For this application, the control inputs to the control actuators are also at the same frequencies. How to effectively generate control forces at the required frequencies and without the spillover of the vibration energy at the other nonrelevant frequencies is one of the most important questions for the design of the control system. If the input voltage is defined as:

(3) $$V(t)=\left\{\begin{array}{cc} V_{o}\sin\omega_{o}t & t\geq 0\\ 0 & t<0 \end{array}\right.,$$

then, according to Equation (1), the transmitted force to the solid base is:

(4) $$F_{T}(t)=b_{p}\dot{x}+k_{p}x-k_{p}(cV-h).$$

In Equation (4), $F_{T}(t)$ is the transmitted force to the base structure and only contributed by the elements (damper, spring, and PZT electrical force) that directly contact the base structure.

In this paper, the sensitivity of the actuator for the input voltage specified in Equation (3) is defined as:

(5) $$S(\omega ,\omega_{o},V_{o})=\frac{{\tilde{F}}_{T}(\omega )}{V_{o}},$$

where $\tilde{F}(\omega )$ is the spectrum of F(t).

## 3. Sensitivity of the Piezoelectric Actuator

A simulation study was conducted to analyze the output characteristics. The parameters of the proof mass and the piezoelectric stack actuator to be used in the simulation are listed in Table 1.

### 3.1. State–Space Model

Equations (1) and (2) can be described by the state−space model. The state vector is defined as:

(6) $$X={\left[x,\dot{x},h\right]}^{T},$$

which is determined by the following state−space equation:

(7) $$\dot{X}=A(X,V,\dot{V}),$$

where

(8) $$A(X,V,\dot{V})=\left[\begin{array}{c} \dot{x}\\ \frac{1}{\left(M_{p}+m_{p}\right)}[-b_{p}\dot{x}-k_{p}x+k_{p}(cV-h)]\\ \alpha \dot{V}-\beta \left|\dot{V}\right|h-\gamma \dot{V}\left|h\right| \end{array}\right].$$

Given a time step Δt, the state vector X(m) at t=mΔt is approximated as:

(9) $$X(m)=X(m-1)+A[X(m-1),V(m-1),\dot{V}(m-1)]\Delta t,$$

where $V(m)=V_{o}\sin(\omega_{o}m\Delta t)$, $\dot{V}(m)=\omega_{o}V_{o}\cos(\omega_{o}m\Delta t)$, $X(0)={\left[0,0,0\right]}^{T}$, and m≥0.

**Figure 2:**
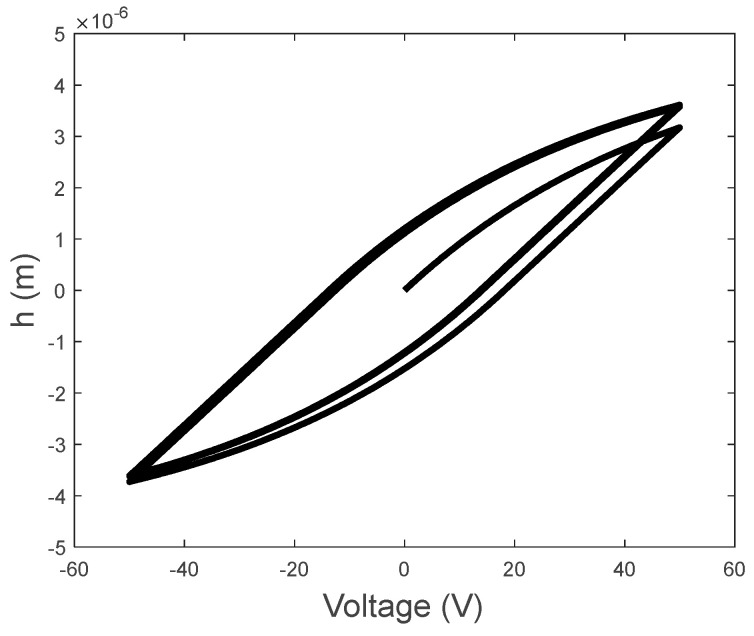
Hysteresis loop of the displacement and applied voltage for $\alpha =1\times 10^{-7}$, β=0.01, and γ=0.01.

**Figure 3:**
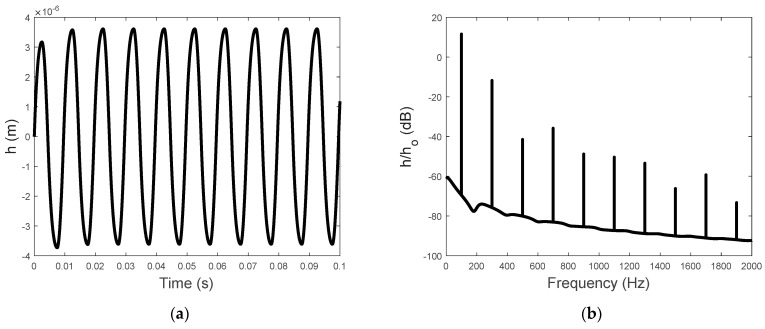
(**a**) The time history and (**b**) the frequency spectrum of the piezoelectric hysteresis displacement for $h_{0}=10^{-6}$ mm.

The numerical calculation in this paper adopted the Newton method. Setting the calculation step dt to 0.00001, the computation of the simulation results was realized by MATLAB 2022a. Using $\omega_{o}=200\pi \;\mathrm{rad}/s$ and $V_{o}=50\;V$, the relationship between the voltage input and the displacement was graphed, as shown in Figure 2, illustrating the hysteresis characteristics in which the displacement is not only dependent on the instantaneous input voltage but also the previous displacement output. The time history of the hysteresis−induced displacement is illustrated in Figure 3a, and the corresponding frequency spectrum is presented in Figure 3b. The spectrum of the hysteresis−induced displacement is characterized by the line spectrum at the driving frequency and its odd harmonics.

It becomes clear that the steady−state driving force on the piezoelectric stack actuator caused by a single−frequency input voltage can be expressed by the Fourier transform of the time−domain driving force as:

(10) $$F_{PZT}(k\omega_{o})=\tilde{F}[k_{p}(cV-h)],\;k=1,3,5,\ldots .$$

Figure 3b also shows that the steady−state hysteresis−induced displacement is dominated by the first two frequency components (k=1,3). The magnitudes of the other components are at least 20 dB below the dominant components. This observation motivated the following analytical derivation of the first four frequency components of $F_{PZT}(k\omega_{o})$ for k=0,1,2,3, as shown in Section 3.2.

### 3.2. Analytical Model

We assume a steady−state input voltage:

(11) $$V(t)=V_{o}\sin\omega_{o}t,\;-\infty \leq t\leq \infty .$$

Inspired by the multiple scale method for the forced vibration of a nonlinear system [20,21], a scaled parameter $T_{r}=\varepsilon^{r}t\;(r=0,1,2\ldots )$ is introduced. By expressing h and the time derivatives as an expansion dependent on the scaled parameter, substituting the expanded series into Equation (2), and equating the coefficients at order $\varepsilon^{0}$, we obtain:

(12) $$D_{0}h_{0}=\alpha D_{0}V.$$

Therefore, taking only the first order of the expansion gives:

(13) $$\left|h\right|\approx \left|h_{0}\right|=\alpha V_{o}\left|\sin\omega_{o}t\right|.$$

This approximation is used for $\left|h\right|$ in the third term of the right−hand side of Equation (2). This approximation makes the analytical evaluation of $\left|h\right|$ possible by describing $\left|\sin\omega_{o}t\right|$ with the product of a periodic pulse−train function and $\sin\omega_{o}t$. Approximation of $\left|h\right|$ with different frequency components makes its analytical calculation impossible, as the period of the pulse−train function is unknown. As a result, Equation (2) is now written as:

(14) $$\dot{h}=\alpha V_{o}\omega_{o}\cos\omega_{o}t-\beta V_{o}\omega_{o}\left|\cos\omega_{o}t\right|h-\gamma \alpha V_{o}^{2}\omega_{o}\cos\omega_{o}t\left|\sin\omega_{o}t\right|.$$

As shown in Appendix A, the Fourier series expansion of terms in Equation (14) preserves the dominant frequency terms at k=1,3, proving that the observed property of $\left|\dot{h}\right|$ is well retained by this approximation. The applicable range of this equation was investigated by a parameter study, and lab experiments were also conducted to verify the results.

By using the following complex exponential expansion:

(15) $$h(t)=\sum_{k=-\infty }^{\infty }h_{k}e^{jk\omega_{o}t}$$

and comparing the coefficients of the complex Fourier series transform on both sides of Equation (14) at each frequency component, while ignoring the contribution of the higher−order frequency components ($\left|k\right|\geq 5$) (see Appendix A for details), the following approximated analytical results are obtained:

(16) $$h_{k}=0\;\mathrm{for}\;k=0,\pm 2,\pm 4,\ldots .$$

For k=±1,±3:

(17) $$h_{\pm 1}=R_{1}\pm I_{1}j\;\mathrm{and}$$

(18) $$h_{\pm 3}=R_{3}\pm I_{3}j,$$

where

(19) $$R_{1}=\frac{AF-DC}{DB-AE},$$

(20) $$I_{1}=\frac{BR_{1}+C}{A},$$

(21) $$R_{3}=\frac{\left(\frac{8\beta \omega_{o}V_{o}}{3\pi })R_{1}-I_{1}\omega_{o}-(\frac{\alpha V_{o}\omega_{o}}{2}-\gamma \alpha V_{o}^{2}\omega_{o}\frac{1}{3\pi }\right)}{-\frac{8\beta \omega_{o}V_{o}}{15\pi }},\;\mathrm{and}$$

(22) $$I_{3}=\frac{R_{1}\omega_{o}+(\frac{4\beta \omega_{o}V_{o}}{3\pi })I_{1}}{-\frac{12\beta \omega_{o}V_{o}}{15\pi }}.$$

In Equations (19) and (20), A,B,C,D,E,F are expressed as:

(23) $$A=-3648{\left(\frac{\beta \omega_{o}V_{o}}{15\pi }\right)}^{3}+\frac{12}{5\pi }\beta \omega_{o}{}^{3}V_{o},$$

(24) $$B=\frac{112\beta^{2}\omega_{o}{}^{3}V_{o}{}^{2}}{15\pi^{2}},$$

(25) $$C=-\frac{12\beta \omega_{o}{}^{2}V_{o}}{5\pi }(\frac{\alpha V_{o}\omega_{o}}{2}-\gamma \alpha V_{o}^{2}\omega_{o}\frac{1}{3\pi }),$$

(26) $$D=\frac{56\beta^{2}\omega_{o}{}^{3}V_{o}{}^{2}}{15\pi^{2}},$$

(27) $$E=13632{\left(\frac{\beta \omega_{o}V_{o}}{15\pi }\right)}^{3}-\frac{8}{5\pi }\beta \omega_{o}{}^{3}V_{o},\;\mathrm{and}$$

(28) $$F=\frac{\beta^{2}\omega_{o}{}^{2}V_{o}{}^{2}}{225\pi^{2}}(696\gamma \alpha V_{o}^{2}\omega_{o}\frac{1}{5\pi }-180\alpha V_{o}\omega_{o}).$$

Therefore, the spectra of the first and third frequency components of the piezoelectric driving force are obtained as:

(29) $$F_{PZT}(\omega_{o})=-j\frac{cV_{o}}{2}-h_{1}\mathrm{and}$$

(30) $$F_{PZT}(3\omega_{o})=-h_{3}.$$

The steady−state displacement of the proof mass due to the spectral components of the piezoelectric driving force (input/output of the second part of the system) is readily derived from the response of a linear oscillator:

(31) $$x_{s}(n\omega_{o})=\frac{F_{PZT}(n\omega_{o})}{k_{p}\{[1-{\left(\frac{n\omega_{o}}{\omega_{n}}\right)}^{2}]+2i\zeta \frac{n\omega_{o}}{\omega_{n}}\}},$$

where $\omega_{n}=\sqrt{\frac{k_{p}}{M_{p}+m_{p}}}$ and $\zeta =\frac{b_{p}}{2\omega_{n}(M_{p}+m_{p})}$ are, respectively, the natural frequency and damping ratio of the actuator. Substituting the steady−state displacement (Equation (31)) and piezoelectric driving force (Equation (10) or Equations (29) and (30) into Equation (5) gives rise to the steady−state sensitivity:

(32) $$S(k\omega_{o},V_{o})=\{\frac{\left(ik\omega_{o}b_{p}+k_{p}\right)}{k_{p}\{[1-{\left(\frac{k\omega_{o}}{\omega_{n}}\right)}^{2}]+2i\zeta \frac{k\omega_{o}}{\omega_{n}}\}}-1\}\frac{F_{PZT}(k\omega_{o})}{V_{o}}.$$

### 3.3. Simulated Results

**Figure 4:**
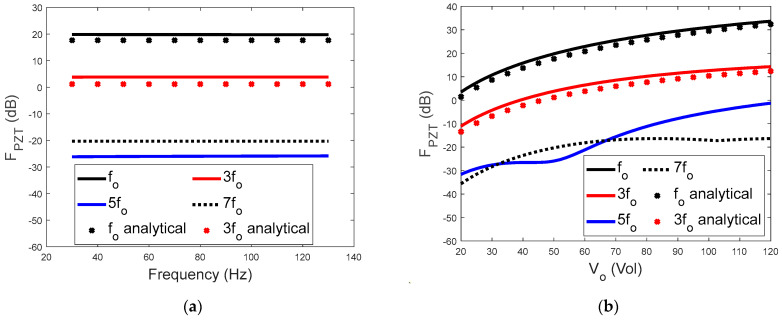
(**a**) Effects of the driving frequency of the input voltage on the first four spectral lines of the piezoelectric driving force with $V_{0}=50\;V$ and (**b**) effects of the amplitude of the input voltage on the first four spectral lines of the piezoelectric driving force with $\omega_{o}=200\pi \;\mathrm{rad}/s$ (β/γ=1).

Figure 4 shows the effect of the frequency and amplitude of the input voltage on the line spectra of the piezoelectric driving force. As shown in Figure 4, four $\left|F_{PZT}(k\omega_{o})\right|$ (*k* = 1, 3, 5, 7) curves were calculated by numerically solving Equation (2). The analytical results from Equations (29) and (30) are also presented in Figure 4 for comparison. It is worth noting that, in Figure 4a, the horizontal axis is the frequency of the input voltage, and the vertical axis is the spectral level of the frequency components. The frequency of each component is illustrated in the legend. Figure 4a shows a large difference in spectral level between the components. For example, $F_{PZT}(\omega_{o})$ is approximately 15 dB higher than $F_{PZT}(3\omega_{o})$, indicating that the force component at the driving frequency of the input voltage has the largest amplitude. This property of the piezoelectric driving force could be utilized for the effective generation of control forces. The amplitudes of the higher−order components are all more than 20 dB greater than that of $F_{PZT}(3\omega_{o})$. Therefore, Figure 4a further demonstrates that the piezoelectric driving force is dominated by its first two frequency components.

The dependence of the piezoelectric driving force on the amplitude of the input voltage is presented in Figure 4b. The increase in $\left|F_{PZT}(k\omega_{o})\right|$ with the voltage amplitude is not linear. Typically, as the amplitude doubles from 50 V to 100 V, $\left|F_{PZT}(\omega_{o})\right|$ increases by approximately 17 dB, whilst $\left|F_{PZT}(3\omega_{o})\right|$ increases by 10 dB.

**Figure 5:**
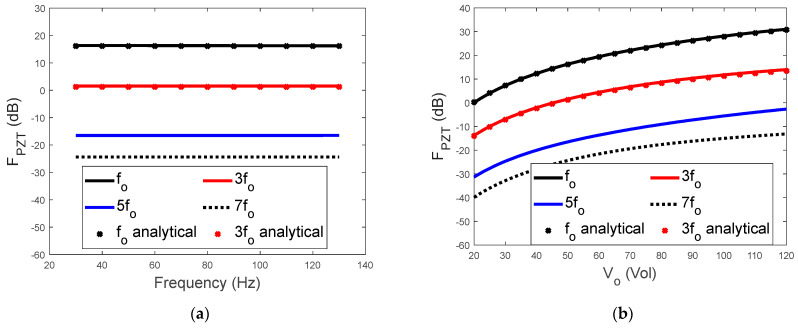
(**a**) Effects of the driving frequency of the input voltage on the first four spectral lines of the piezoelectric driving force with *V_0_* = 50 V and (**b**) effects of the amplitude of the input voltage on the first four spectral lines of the piezoelectric driving force with $\omega_{o}=200\pi \;\mathrm{rad}/s$ (β/γ=10).

Figure 4 also shows that the approximated analytical results for $\left|F_{PZT}(k\omega_{o})\right|$ (*k* = 1, 3) are reasonably comparable with those from the numerical solution. Further parametric analysis indicates that the accuracy of the analytical results is dependent on the ratio between β and γ. When β/γ=1, which is the case shown in Figure 4, the differences between the analytical and numerical results are still observable. When β/γ=10, as shown in Figure 5, the agreement between the analytical and numerical results improved significantly.

**Figure 6:**
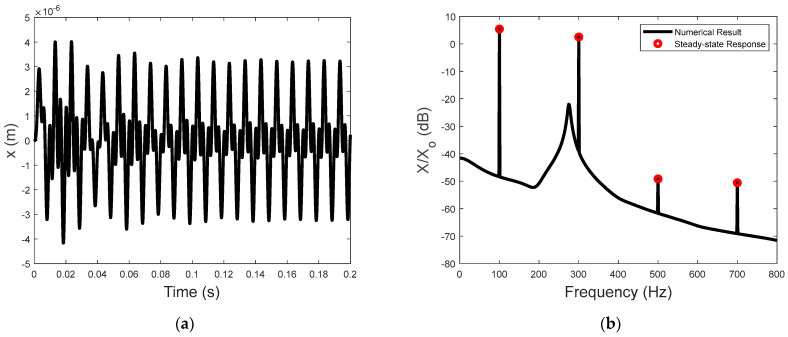
(**a**) The time−domain displacement of the proof mass due to the single−frequency input voltage at 100 Hz and 50 V and (**b**) the frequency−domain displacement of the proof mass ($x_{o}=10^{-6}\;m$).

The time history of the displacement of the proof mass and the corresponding frequency spectrum are presented in Figure 6. The non−sinusoidal displacement in Figure 6a is due to the excitation of the piezoelectric driving force at multiple frequencies. As expected, the displacement spectrum shown in Figure 6b is also characterized by the displacement components at the driving frequency and its odd harmonics. The analytical spectral level of the steady−state displacement compares well with the numerical results, indicating that the steady−state displacement can be understood using the simple analytical expression shown in Equation (26). The resonance peak of the actuator is also observable in the background spectrum of the displacement response.

**Figure 7:**
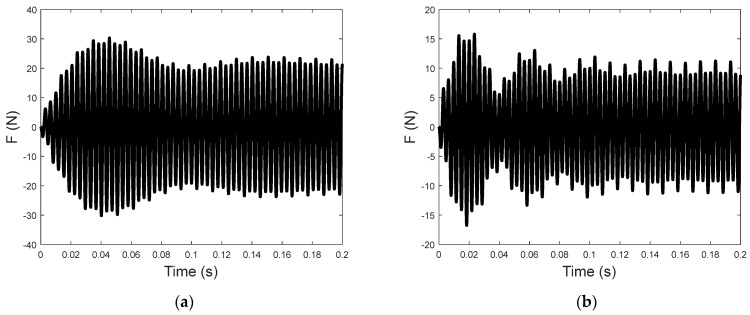
The time−domain transmitted force due to the input voltage (**a**) at 95 Hz and (**b**) at 100 Hz.

**Figure 8:**
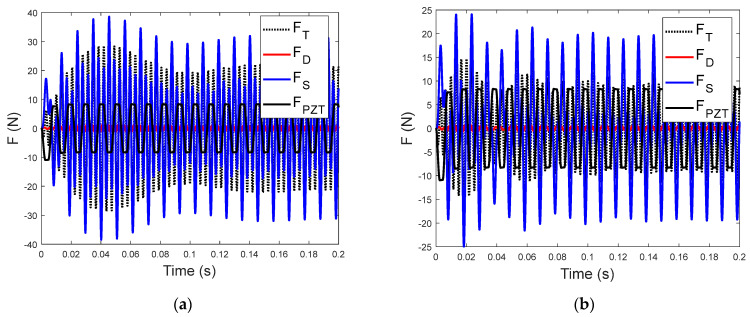
Components of the transmitted force for (**a**) a driving frequency of 95 Hz and (**b**) voltage excitation at 100 Hz.

To illustrate the trends of the sensitivity, the time histories of the transmitted force are shown in Figure 7 for input voltages at two different driving frequencies. The time−domain transmitted force for *f*_0_ = 95 Hz, as shown in Figure 7a, shows a transient rise in the early part of the oscillation, while that for *f*_0_ = 100 Hz (see Figure 7b) has a beating−like vibration in the transient displacement. As the transmitted force includes the damping ($F_{D}$), stiffness ($F_{S}$), and piezoelectric ($F_{PZT}$) force components, the time history of the transmitted force can be explained by the superposition of the force components. Figure 8 shows that the transmitted forces are dominated by the stiffness force component, although the full properties of the transient and steady−state time histories of the total transmitted force are still due to the superposition of all the components.

In Figure 8a, the stiffness force has a strong transient component, which results in a similar transient in the total transmitted force. The beating−like transient behavior (the period of the beating is approximately 0.04 s) shown in Figure 8b may be explained by the superposition of the free vibration components at the natural frequency of 275 Hz and the forced vibration component at 300 Hz. Nonetheless, the transient behavior of the transmitted force is determined by the initial conditions of the proof mass and the hysteresis−induced displacement, the transient feature of the input voltage, and the system dynamics. Although the transient response is a very small portion of the energy in the sampled data, it consists of broad−band frequency components and system resonance that can be observed in the background spectrum of the sensitivity.

**Figure 9:**
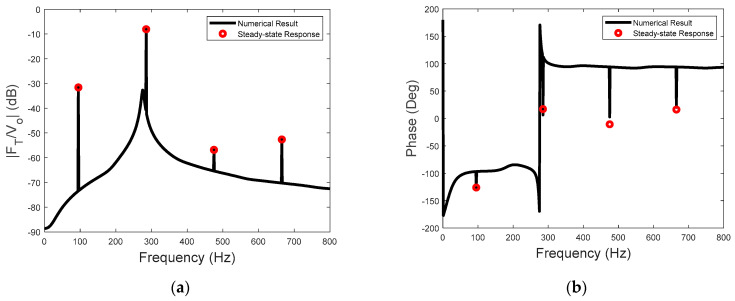
(**a**) The sensitivity of the actuator and (**b**) the phases of the steady−state sensitivity due to the voltage excitation at 95 Hz.

The sensitivity of the actuator for input voltage at 95 Hz and 50 V is presented in Figure 9. Also included are the line spectral levels and phases of the steady−state sensitivity calculated using Equation (27). As the third harmonic frequency of the piezoelectric driving force is close to the natural frequency of the actuator, the resonance−enhanced transmitted−force spectrum is clearly observable at this frequency (see Figure 9a). Thus, Equation (27) can be used for achieving maximum sensitivity, which involves setting the driving frequency of the input voltage close to the natural frequency of the actuator system. The phases of the steady−state sensitivity are shown in Figure 9b, which include the phases of the piezoelectric driving force and the linear dynamics of the actuator. The broad−band phase spectrum is also shown in Figure 9b, with a large phase jump at the natural frequency of the actuator. However, the phase of the sensitivity presented in Figure 9b is only the phase of the one−sided spectrum in Equation (5). It is not the traditional frequency response function of a linear input and output system, as the input voltage only has a steady−state component at a single frequency. Nevertheless, if the relative phase is to be considered, then the phase of the piezoelectric force in Equation (10) could be used as the reference.

**Figure 10:**
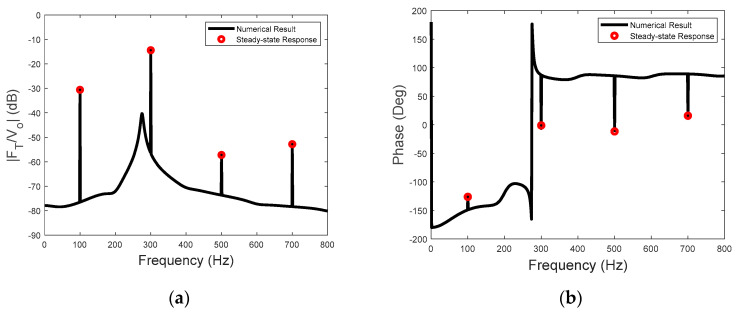
(**a**) The sensitivity of the actuator and (**b**) the phases of the steady−state sensitivity due to the voltage excitation at 100 Hz.

The sensitivity to the input voltage at 100 Hz and 50 V is shown in Figure 10. Again, the steady−state amplitude and the phase of the sensitivity can be accurately predicted by the analytical solution of the two−stage model. Moreover, the system resonance is observable in both Figure 9b and Figure 10b because of the transient part of the transmitted forces.

## 4. Experimental Validation

**Figure 11:**
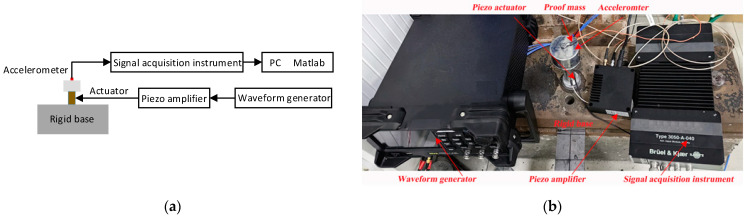
Experiment for obtaining the vibration of a piezoelectric stack actuator: (**a**) block diagram; (**b**) experimental equipment and setup.

To validate the proposed theory and results, an experimental setup for obtaining the vibration of a piezoelectric stack actuator was developed, as shown in Figure 11. The test piezoelectric stack (CoreMorrow, PSt150/10/60 VS15) was fixed on a rigid base structure on the bottom. The mass of the test piezoelectric stack was determined by the longitudinal response frequency of the stack when the proof mass was not attached. A sinusoidal voltage signal from a waveform generator (Rigol, DG4000) was amplified via a controller (CoreMorrow, E53.A). The vibration of the proof mass (1000 g) was measured by an accelerometer (B&K, 4517−002). The output of the accelerometer was pre−amplified by a signal acquisition instrument (B&K, 2207) before being sent to a laptop computer for postprocessing. It should be noted that the accelerometer used in the experimental measurement must be lightweight to reduce the impact of its mass on the system. Thus, the accelerometer (B&K, 4517−002), with 0.7 g weight, was a good choice in the test.

**Figure 12:**
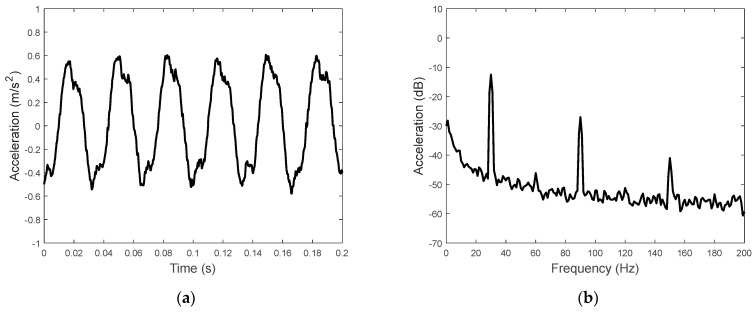
Acceleration on the top of the proof mass: (**a**) time domain and (**b**) frequency domain.

For this case, the signal acquisition instrument was used for data acquisition and calculating the time− and frequency−domain data of the accelerations. With a sinusoidal excitation voltage at 30 Hz and 45 V, the acceleration signal is shown in Figure 11. The non−sinusoidal acceleration shown in Figure 12a is due to the excitation of the piezoelectric driving force at multiple frequencies. The acceleration spectrum shown in Figure 12b is also characterized by the acceleration components at the driving frequency and its odd harmonics.

This result was used for parameter identification of the model. It is worth noting that the Bouc−Wen model is a phenomenological model, and the parameters α, β, and γ in Equation (2) do not necessarily have a physical meaning. Instead, two physical constraints are usually implemented while using the model [22]: the second law of thermodynamics is fulfilled if and only if β>0 and −β≤γ≤β; the model is bounded−input−bounded−output stable and consistent with the motion of physical systems if α>0, β+γ>0, and β−γ≥0. These conditions ensure the physical and mathematical consistency of the Bouc−Wen model.

To ensure the model consistency, the conditions in Table 2 were adopted for the parameter identification of the model. Using the particle swarm optimization (PSO) method [23], the parameters in Equations (1) and (2) were identified as $k_{p}=3.61\times 10^{6}\;N/m$, $b_{p}=40.77\;\mathrm{Ns}/m$, $c=0.3653\times 10^{-6}\;m/V$, $\alpha =0.2192\times 10^{-6}$, β=0.0214, and γ=0.0037. These values were used in the calculation of the analytical and numerical model.

**Figure 13:**
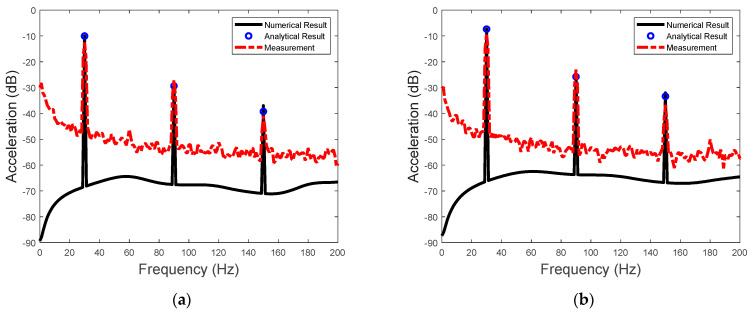
The frequency−domain acceleration of the proof mass excited by different voltages: (**a**) sinusoidal excitation voltages at 30 Hz and 45 V and (**b**) sinusoidal excitation voltages at 30 Hz and 60 V.

As Figure 13 shows, the acceleration spectrum can be characterized by the acceleration components at the driving frequency and its odd harmonics. This phenomenon is also consistent with the displacement spectrum shown in Figure 6b. Figure 13a shows that $Acc(\omega_{o})$ is approximately 17 dB higher than $Acc(3\omega_{o})$. The overall error between the experimental results and the analytical and numerical results is less than 1 dB at 30 Hz. In addition, the increase in $\left|Acc(k\omega_{o})\right|$ with the voltage amplitude is not linear. As the amplitude increases from 45 V to 60 V, $\left|Acc(\omega_{o})\right|$ increases by approximately 3.7 dB, whilst $\left|Acc(3\omega_{o})\right|$ increases by 1.3 dB.

**Figure 14:**
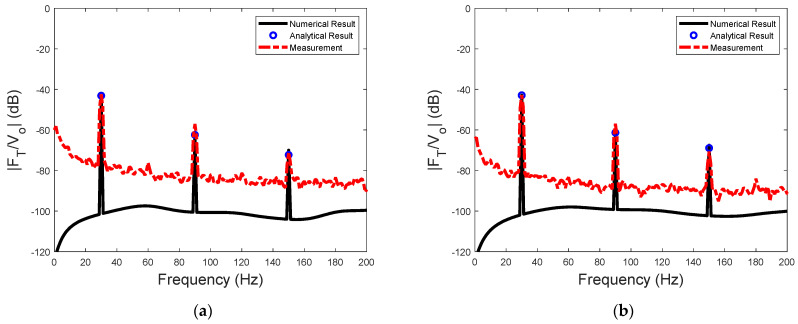
The sensitivity of the actuator excited by different voltages: (**a**) sinusoidal excitation voltages at 30 Hz and 45 V and (**b**) sinusoidal excitation voltages at 30 Hz and 60 V.

Using acceleration data, the sensitivity of measurement can be obtained from Equations (1) and (5). The sensitivity of the actuator for input voltage at 30 Hz, 45 V, and 60 V is presented in Figure 14. Again, the steady−state amplitude can be accurately predicted by the analytical solution of the two−stage model. The harmonic components in the spectrum of the sensitivity predicted by the model could match the measured data well.

## 5. Conclusions

Owing to the nonlinear hysteresis effect, the nonlinear input/output relationship of the actuator becomes input−dependent. In this paper, the sensitivity of the actuator was defined by a single−frequency input voltage as the input and the spectrum of the transmitted force by the actuator as the output. This steady−state analysis of the sensitivity illustrated that the nonlinear dynamics of the actuator can be interpreted as a cascade of two subsystems. The first subsystem includes the nonlinear hysteresis that transforms a single−frequency input voltage into a piezoelectric driving force at the driving frequency and its odd harmonics, while the second is a linear subsystem excited by the driving force.

The numerical solution of the hysteresis−induced displacement in the actuator revealed the steady−state property of the first subsystem. The dynamics of the second subsystem could be described analytically by the frequency response functions at the frequency of the input voltage and its odd harmonics. As a result, the steady−state displacement of the proof mass and the transmitted force were obtained in simple analytical expressions whose amplitude and phase characteristics could be used for the design and modeling of the piezoelectric stack actuator.

Analytical expressions of the steady−state piezoelectric driving force at the frequency of input voltage and its third harmonic frequency were also derived approximately. The derivation explained why the piezoelectric force components at the even harmonic frequencies of the input voltage were zero.

Finally, an experiment was implemented to verify the steady−state features of the model in describing the hysteresis of the piezoelectric stack actuator attached with a proof mass. The results showed that the harmonic components in the spectrum of the vibration acceleration predicted by the model could match the measured data well.

In future work, it will be necessary to discuss how to suppress the spillover effect of the actuator at the frequencies due to the nonlinear mapping from the single−frequency input voltage to the piezoelectric force at multiple frequencies. Other configurations of the actuator should also be considered in order to avoid the use of a heavy proof mass.

## Figures and Tables

**Table 1:** Parameters used for modeling the piezoelectric stack actuator.

Parameters	Value
$M_{p}$	2.0 kg
$m_{p}$	0.004 kg
$b_{p}$	150 Ns/m
$k_{p}$	$6\times 10^{6}$ N/m
c	$1\times 10^{-7}$ m/V
α	$1\times 10^{-7}$
β	0.01
γ	0.01

**Table 2:** Conditions of the parameters in the Bouc–Wen model.

**Parameters**	α	β	γ
Conditions	α>0	β > 0	β + γ > 0 and β − γ ≥ 0

## Data Availability

The data presented in this study are available on request from the corresponding author. The data are not publicly available due to a confidentiality agreement for the project.

## Appendix A

The periodic properties of the first and third terms on the right−hand side of Equation (14) indicate that the steady−state property of h(t) is also a periodic time function with a period of $T=\frac{2\pi }{\omega_{o}}$ and can be expanded by complex exponential functions:

(A1) $$h(t)=\sum_{k=-\infty }^{\infty }h_{k}e^{jk\omega_{o}t}.$$

The solution of the coefficients $h_{k}$ may be carried out by applying a complex Fourier series transform on Equation (14) and equating the coefficients of the same frequency component on the two sides of the resulting equation. The advantage of using this method of Fourier series transform is that absolute periodic functions, such as $\left|\cos\omega_{o}t\right|$ and $\left|\sin\omega_{o}t\right|$ in Equation (14), can be calculated by the convolution of a Fourier−transformed pulse−train function and the periodic function. For example:

(A2) $$\left|\cos\omega_{o}t\right|=p_{T}(t)\cos\omega_{o}t,$$

where $p_{T}(t)$ is a rectangular pulse−train function:

(A3) $$p_{T}(t)=\left\{\begin{array}{cc} 1, & 0\leq t\leq \frac{T}{4},\frac{3T}{4}\leq t\leq T\\ -1, & \frac{T}{4}\leq t\leq \frac{3T}{4} \end{array}\right..$$

Thus, the Fourier transform of $\left|\cos\omega_{o}t\right|$ is implemented as:

(A4) $${\tilde{F}}_{T}\{\left|\cos\omega_{o}t\right|\}={\tilde{p}}_{T}(k)*{\tilde{F}}_{T}\{\cos\omega_{o}t\}=\left\{\begin{array}{cc} -\frac{2{\left(-1\right)}^{\frac{k}{2}}}{\pi \left(k^{2}-1\right)} & k\;=0,\pm 2,\pm 4\\ 0 & k\;=\pm 1,\pm 3,\ldots \end{array}\right.$$

where “∗” represents convolution. Similarly, the Fourier transform of $\left|\sin\omega_{o}t\right|$ is obtained as

(A5) $${\tilde{F}}_{T}\{\left|\sin\omega_{o}t\right|\}==\left\{\begin{array}{cc} -\frac{1}{\pi \left(k^{2}-1\right)} & k\;=0,\pm 2,\pm 4,\ldots \\ 0 & k\;=\pm 1,\pm 3,\ldots \;. \end{array}\right.$$

Furthermore, the Fourier transform of $\left|\cos\omega_{o}t\right|h$:

(A6) $${\tilde{F}}_{T}\{\left|\cos\omega_{o}t\right|h\}={\tilde{F}}_{T}\{\left|\cos\omega_{o}t\right|\}*{\tilde{F}}_{T}\{h\}=\sum_{l=0}^{\infty }a_{2l}\sum_{k\prime =-\infty }^{\infty }h_{k\prime }\left[e^{j(k\prime +2l)\omega_{o}t}+e^{j(k\prime -2l)\omega_{o}t}\right],$$

where

(A7) $$a_{2l}=\left\{\begin{array}{cc} -\frac{1}{\pi } & l=0\\ \frac{2{\left(-1\right)}^{l}}{\pi \left({\left(2l\right)}^{2}-1\right)} & l>0\;, \end{array}\right.$$

and

(A8) $${\tilde{F}}_{T}\{\cos\omega_{o}t\left|\sin\omega_{o}t\right|\}={\tilde{F}}_{T}\{\cos\omega_{o}t\}*{\tilde{F}}_{T}\{\left|\sin\omega_{o}t\right|\}=\left\{\begin{array}{cc} -\frac{1}{\pi \left(k^{2}-4\right)} & k=\pm 1,\pm 3,\ldots \\ 0 & k\;=0,\pm 2,\pm 4,\ldots \;. \end{array}\right.$$

To determine $h_{k}$, the coefficients of the terms with each frequency component on both sides of Equation (14) are listed in Table A1. Equating the coefficients of the two sides gives rise to algebraic equations for k=0,±1,±2,±3. As amplitudes of $h_{k}$ with k=±4,±5,±6,… are much smaller than those of $h_{\pm 1}$ and $h_{\pm 3}$, the variables of the algebra equations are truncated from $\left|k\right|\geq 4$.

**Table A1:** Fourier series transform coefficients of the terms in Equation (14).

$\dot{h}$	$\alpha V_{o}\omega_{o}\cos\omega_{o}t$	$-\beta V_{o}\omega_{o}\left|\cos\omega_{o}t\right|h$	$-\gamma \alpha V_{o}^{2}\omega_{o}\cos\omega_{o}t\left|\sin\omega_{o}t\right|$
0	0	$a_{0}h_{0}+a_{2}h_{2}+a_{2}h_{-2}$	0
$\pm j\omega_{o}h_{\pm 1}$	$b_{\pm 1}=\frac{\alpha V_{o}\omega_{o}}{2}$	$a_{0}h_{\pm 1}+a_{2}h_{\pm 3}+a_{2}h_{\mp 1}+a_{4}h_{\mp 3}$	$c_{\pm 1}=-\gamma \alpha V_{o}^{2}\omega_{o}\frac{1}{3\pi }$
$\pm j2\omega_{o}h_{\pm 2}$	0	$a_{0}h_{\pm 2}+a_{2}h_{0}+a_{4}h_{\mp 2}$	0
$\pm j3\omega_{o}h_{\pm 3}$	0	$a_{0}h_{\pm 3}+a_{2}h_{\pm 1}$	$c_{\pm 3}=\gamma \alpha V_{o}^{2}\omega_{o}\frac{1}{5\pi }$

The second and fourth rows of Table A1 yield the following matrix equation:

(A9) $$\left[\begin{array}{ccc} a_{0} & a_{2} & a_{2}\\ a_{2} & a_{4} & a_{0}-j2\omega_{o}\\ a_{2} & a_{0}+j2\omega_{o} & a_{4} \end{array}\right]\left[\begin{array}{c} h_{0}\\ h_{-2}\\ h_{2} \end{array}\right]=0.$$

Using Equation (A7), it can be shown that:

(A10) $$\det\left[\begin{array}{ccc} a_{0} & a_{2} & a_{2}\\ a_{2} & a_{4} & a_{0}-j2\omega_{o}\\ a_{2} & a_{0}+j2\omega_{o} & a_{4} \end{array}\right]=\frac{4\omega_{o}^{2}}{\pi }-\frac{17}{675\pi^{3}}.$$

Thus, the coefficient matrix of Equation (A9) has full rank unless $\omega_{o}^{}=\frac{1}{10\pi }\sqrt{\frac{17}{27}}$, which corresponds to a very low frequency of 0.0253 rad/s. This indicates that in the frequency of interest:

(A11) $$h_{0}=h_{\pm 2}=0.$$

The third and fifth rows of Table A1 result in the following matrix equation:

(A12) $$\left[\begin{array}{cccc} a_{0}+j\omega_{o} & a_{2} & a_{2} & a_{4}\\ a_{2} & a_{0}-j\omega_{o} & a_{4} & a_{2}\\ a_{2} & a_{4} & a_{0}+j3\omega_{o} & 0\\ a_{4} & a_{2} & 0 & a_{0}-j3\omega_{o} \end{array}\right]\left[\begin{array}{c} h_{-1}\\ h_{1}\\ h_{-3}\\ h_{3} \end{array}\right]=-\left[\begin{array}{c} b_{-1}+c_{-1}\\ b_{1}+c_{1}\\ c_{-3}\\ c_{3} \end{array}\right].$$

The analytical solutions of ${\left[\begin{array}{cccc} h_{-1} & h_{1} & h_{-3} & h_{3} \end{array}\right]}^{T}$ are obtained as:

(A13) $$h_{\mp 1}=R_{1}\mp jI_{1}\;\mathrm{and}$$

(A14) $$h_{\mp 3}=R_{3}\mp jI_{3},$$

where functions $R_{1}$, $I_{1}$, $R_{3}$, and $I_{3}$ have already been presented in Equations (19)–(22).
